# COVID-19 impact on overweight and obesity rates in Aotearoa | New Zealand 4-year-old children

**DOI:** 10.1038/s41390-024-03025-6

**Published:** 2024-01-18

**Authors:** Philip J. Schluter, Annabel Ahuriri-Driscoll, Jalal Mohammed, Sheetalpreet Singh

**Affiliations:** 1 Te Kaupeka Oranga, Faculty of Health, Te Whare Wānanga o Waitaha, University of Canterbury, Christchurch, Aotearoa New Zealand; 2 School of Clinical Medicine, Primary Care Clinical Unit, The University of Queensland, Brisbane, QLD Australia; 3 Service Analysis and Modelling Evidence, Research and Analytics Evidence Research and Innovation, Ministry of Health, Wellington, Aotearoa New Zealand

## Abstract

**Background:**

COVID-19 has had profound societal impacts. This study estimated overweight, obesity, and extreme obesity rates in 4-year-old children over pre- and post-COVID-19 periods, and investigated differential changes between sex, ethnic and deprivation groups.

**Methods:**

A national screening programme of 4-year-old children undertaking B4 School Checks (B4SCs) between 1 January 2010 and 7 March 2023 was analysed. B4SCs include anthropometric measurements enabling sex-specific body mass index-for-age Z-scores (BMI z-scores) to be derived. Children with ≥85th, ≥95th, and ≥99.7th percentile BMI z-scores were classified as overweight, obese, and extremely obese.

**Results:**

The eligible sample included 656,038 children (48.8% girls). Overall, 210,492 (32.1%) children were overweight, 95,196 (14.5%) obese, and 19,926 (3.0%) extremely obese. While decreasing in the pre-COVID-19 period, annual prevalence estimates for overweight, obese, and extremely obese significantly (all *p* < 0.001) increased in the year after COVID-restrictions were implemented. However, after three years, overweight and obese prevalence estimates were no different to pre-COVID levels overall or stratified by sex for ethnicity and deprivation groups. Extreme obesity prevalence estimates also decreased but remained higher than pre-COVID levels.

**Conclusion:**

The sharp and steep increases in prevalence estimates all dampened relatively quickly. The question remains whether these rates will continue to decrease in time.

**Impact:**

- Compared to pre-COVID-19 estimates, the prevalence of overweight, obesity and extreme obesity significantly and substantially increased for 4-year-old children in the immediate post-COVID-19 period.
- These post-COVID-19 prevalence estimates dampened relatively quickly, returning to pre-COVID-19 rates for overweight and obesity after 3 years.
- Inequities between ethnic and social deprivation groups in overweight and obesity prevalence estimates remained similar between pre- and post-COVID-19 periods.

## Introduction

The global prevalence of obesity is estimated to exceed 1 billion people, includes 340 million adolescents and 39 million children, and continues to increase.^1^ Obesity is a complex and multifaceted health condition that can have a profound impact on various body systems and leads to a range of non-communicable diseases, as well as mental health issues.^1^ It also carries a high societal and economic cost, irrespective of the geographical context.^2^ Correspondingly, the global burden of disease attributable to high body mass index (BMI) is worsening over time, exacerbating an already major global health challenge.^3^ It is recognised that the key to preventing obesity is to act early in young children.^1^

The novel coronavirus disease 2019 (COVID-19) outbreak, declared by the World Health Organization (WHO) as a global pandemic on 11 March 2020, has had far-reaching impacts on all facets of society.^4,5^ Soon after this announcement, many countries introduced stringent public health restrictions, including lockdowns, quarantine periods, and stay-at-home orders. These restrictions, and the COVID-19 aftereffects, led to behavioural and lifestyle changes associated with increased obesity risk (including physical inactivity, poor diet, stress, and poverty) for many people.^6^ A recent systematic review of 74 longitudinal studies, which included 31 studies of children, 41 studies of adults, and 2 studies of children and adults, found increased prevalence of obesity in both children and adults within the post-COVID-19 period.^7^ Increases were found to be greater in children, and targeted prevention interventions were advocated.

Within Aotearoa | New Zealand (ANZ), the childhood obesity epidemic forms a core component of the National Science Challenge: A Better Start | E Tipu e Rea.^8^ The National Science Challenges, established and funded by Government since 2014, aim to tackle the biggest science-based issues and opportunities facing ANZ.^9^ Perhaps due, in part, to this investment, and other population health contributions,^10^ two studies using data from a national screening programme (the B4 School Check; B4SC) have shown declining rates of overweight, obesity and extreme obesity among 4-year-old children in ANZ during the pre-COVID-19 periods of 2010-2016 and 2017-2019.^10,11^ Moreover, downward trends were observed across sex, ethnicity and deprivation groups. The B4SC is the eighth and final contact point within ANZ’s Well Child Tamariki Ora program, which is available cost-free to all families of children aged from 6 weeks to 5 years. The B4SC screens 4-year-old children with a focus on identifying any social, developmental or behavioural issues which could potentially interfere with children’s learning and success at school.^12^ Within this pre-COVID-19 period, B4SC uptake was high with population coverage exceeding 90% since 2013.^11^

Like many countries around the world, to mitigate the impact of COVID-19, ANZ rapidly implemented strict public health measures. These included closing its borders to non-nationals, introducing lockdowns and contact tracing, and restricting movement and interactions between people.^13^ Early childhood centres were closed, as were playgrounds, and contact beyond immediate family and households heavily curtailed. Non-essential services were also paused or postponed, including B4SC assessments.

While it was encouraging that rates of overweight, obesity and extreme obesity in ANZ 4-year-old children decreased in the pre-COVID-19 period, it is of interest to explore whether these trends were sustained or interrupted by the introduction of public health restrictions. Also, although most restrictions have now been lifted, it is unknown whether the pandemic will have enduring detrimental impacts on health behaviours, potentially impeding or reversing the progress made in reducing obesity prevalence during the pre-COVID-19 era.^7^ Thus, this study aimed to: investigate rates of overweight, obesity and extreme obesity in 4-year-old children before and after ANZ Government’s COVID-19 pandemic response was implemented; examine whether any changes were different between sex, ethnicity and deprivation groups; and, determine whether any changes in the sociodemographic profile for those completing B4SC assessments post-COVID-19 compared to pre-COVID-19 made a material impact on these prevalence rates.

## Methods

### Study design

A cross-sectional analysis of ANZ 4-year-old children undertaking a B4SC assessment between 1 January 2010 and 7 March 2023, as part of a national screening programme.

### Participants

ANZ children aged between 4 years and 5 years 7 days undertaking their B4SC assessment. Children with valid sex, age and anthropometric measurements were included. However, those found to have biologically implausible sex-specific BMI-for-age Z-scores (BMI z-scores)^14^ were excluded.

### Procedure

A comprehensive description of B4SC procedures is detailed elsewhere.^12,15^ It is a nationwide programme, offered cost-free to parents/caregivers, and includes a wide-ranging set of health and development checks for children aged 4 years. In brief, after receiving informed written consent, B4SC assessments which include anthropometric measurements are conducted by trained registered nurses or nurse practitioners in various community locations and normally take between 45–60 minutes to complete. If concerns are identified, information is offered and support provided which include clinical pathways and referral processes.^15^ The B4SC National Information System within the Ministry of Health houses data relating to the child, permission, assessments and checks, issues identified, and any referrals made. This system is designed to provide non-identifiable information for monitoring the performance of the B4SC programme, for tracking the population health status of 4-year-olds, and for approved research studies.^15^ Population coverage is high, with an estimated 79% of eligible 4-year-olds attending a B4SC in the 2011/12 fiscal year (from July 1 to June 30), 80% in 2012/13, 91% in 2013/14, 92% in 2014/15, 92% in 2015/16, 94% in 2016/17, 93% in 2017/18, and 91% in 2018/19.^10^ After ethics clearance and Ministry of Health application and approval, anonymous unit record data were released for the variables described below.

### Primary measures

After receiving training and a handbook outlining best-practice protocols, nurses or practitioners undertake a variety of assessments.^15^ Anthropometric measurement protocols include measuring the children while they were wearing light clothing with shoes removed, and having the equipment stable on a levelled hard surface. Height is measured to the nearest 0.1 cm using a portable stadiometer (either Leicester Height Measure or a SECA 214) and weight to the nearest 0.1 kg using scales (SECA 862 electronic floor scale; Tanita WB 100S MA floor scale; or, SECA 770 or Tanita HD-351 weighing scale calibrated at least once every 6 months).^11^

Based on the methodology jointly recommended by WHO and UNICEF,^16^ the updated WHO Anthro Macro for Stata software was used to obtain the WHO growth standards for BMI z-scores.^14^ Children with BMI z-scores < –5 or >5 were flagged as being biologically implausible and were excluded. Aligned with previous ANZ studies,^10,11^ children greater than or equal to the 85th, 95th and 99.7th percentile for BMI z-scores were classified as overweight, obese and extremely obese, respectively. The overweight category includes those classified as obese and extremely obese; and the obese category includes those classified as extremely obese. While International Obesity Task Force standards are recommended for intercountry comparisons,^17,18^ this paper focusses on changes over time within ANZ and was deliberately designed to align with previous research.^10,11^.

As part of the ANZ Government’s response to the COVID-19 pandemic, on the 21 March 2020 it introduced a 4-tiered Alert Level system aimed initially at eliminating the virus.^19^ Depending on community case numbers, scientific knowledge, and control measures effectiveness, Alert Levels could increase or decrease, and be different between various geographical regions. Four days after this announcement, ANZ, as a nation, moved to Alert Level 4 and into lockdown. All travel (including local), early childhood centres, gatherings, workplaces, and services were heavily restricted, and individual and societal uncertainty was high.^19^ For the purposes of these analyses, B4SC assessment time was partitioned in years before and after 21 March 2020 with: 21 March 2020–20 March 2021 labelled “1”; 21 March 2021–20 March 2022 labelled “2”; etc.; and, 21 March 2019–20 March 2020 labelled “-1”; 21 March 2018–20 March 2019 labelled “–2”; etc.

### Sociodemographic variables

All sociodemographic characteristics were derived from the B4SC dataset. Sex was categorised as female or male. Age was calculated from B4SC assessment and birth dates. Ethnicity was based on parental/caregiver report, which allows for multiple identifications.^20^ Using the total response approach, whereby categories are not mutually exclusive and individuals could belong to multiple groups, ethnicity was categorised into: Māori, Pacific, Asian, Middle Eastern/Latin American/African (MELAA), European, and Other groups. Māori are the descendants of the indigenous inhabitants of ANZ, while the Other group is comprised of people or descendants from nationalities and ethnicities outside the Pacific, Asian, European, and MELAA regions. Depending on children’s B4SC assessment dates, the New Zealand Deprivation Index 2006 (NZDep2006) or the New Zealand Deprivation Index 2013 (NZDep2013) were used to define deprivation level. For each meshblock (the smallest geographic unit which statistical data are collected and processed by Statistics New Zealand) these indices combine 2006 or 2013 census data, respectively, relating to income, home ownership, employment, qualifications, family structure, housing, access to transport and communications into a single measure.^21^ The measures are then splits into quintiles defined by 1 (least deprived) through to 5 (most deprived) scores. Each child’s recorded residential address at their B4SC assessment was used to determine their meshblock and associated area-level deprivation score. For B4SC assessments prior to 1 October 2015, NZDep2006 was used, otherwise NZDep2013 was employed. Finally, over the study period, health care delivery in ANZ was geographically divided into 20 District Health Boards (DHBs). Each DHB was responsible for providing health services within their district. Geographically contiguous DHB were grouped into four regions, namely: Northern, Midland, Central and Southern. There is substantial variation in both socioeconomic status and ethnicity across these regions,^21,22^ together with regional variations in COVID-19 control measures.^19^

### Statistical analysis

Reporting of analyses were informed by the RECORD guidelines.^23^ Participant flow and sociodemographic characteristics were initially described, as were the B4SC assessment frequencies over time. BMI and BMI z-scores were then presented and classified into overweight, obese and extremely obese groupings. Yearly overweight, obese, and extreme obese prevalence estimates around 21 March 2020 were then presented, and piece-wise linear regression estimates, weighted by participant assessment numbers, with change point constrained at 21 March 2020 were undertaken for the overall eligible sample and stratified by sex. Comparisons of prevalence estimates between periods –1 and 1, and between periods –1 and 3, were undertaken using Students t-tests with unequal variance assumptions. Finally, when investigating the influence of changing sociodemographic patterns between periods –1 and 3, modified Poisson regression models, with robust variance estimators, stratified by sex were undertaken.^24,25^ Adjusted models included age, ethnicity, level of deprivation, region, and all associated two-factor interactions of these terms. Due to the large sample size, and in the spirit of Sun and colleagues,^26^ these adjusted regression models were analysed without any variable selection. All analyses were performed using Stata SE version 17.0 (StataCorp, College Station, TX), and two-tailed α = 0.05 defined significance.

## Results

### Participants

**Fig. 1:**
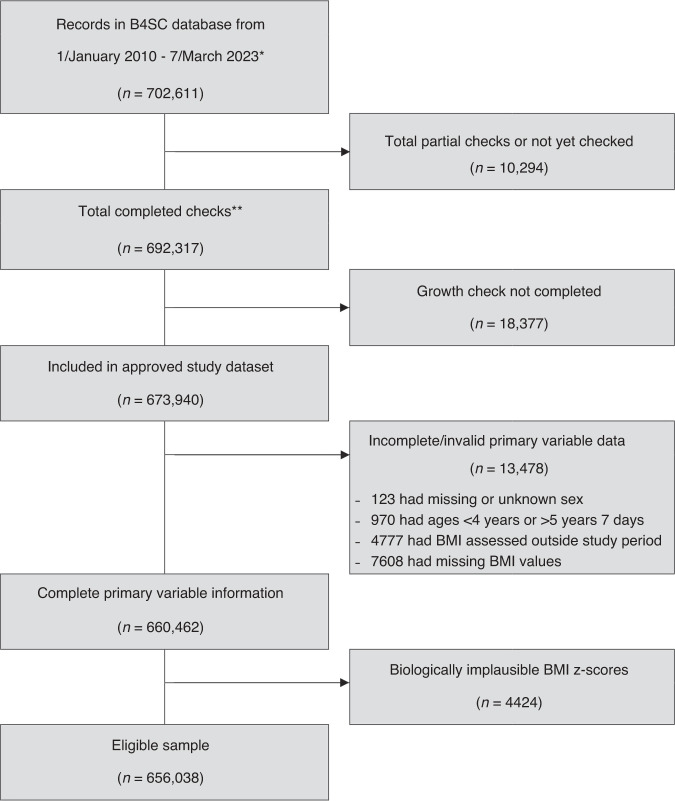
Participant flowchart. Notes: *sourced from the “Completed Closed Assigned” extract and reflects the total number of records where the status of the check is either “Completed”, “Closed”, “Assigned” or “Returned” (does not include records of children who have not yet been assigned to a B4SC provider); **a check is classified as completed if the child’s National Health Index (NHI) has a current status of “Complete” or “Closed” (the NHI is a unique identifier that is assigned to every person who uses the health and disability support services in ANZ) and the NHI has an assigned date first completed while the child is between 4 years and 5 years 7 days.

Data extraction was undertaken by the Ministry of Health on 8 March 2023, with 673,940 records released. However, some anomalies were found with 123 children having either missing or unknown sex, 970 had ages outside the 4 years to 5 years and 7 days interval, 4777 had assessment dates outside the study period interval, 7608 had missing BMI values (due to parent/caregiver or child declining the assessment), and 4424 had biologically implausible BMI z-scores – leaving 656,038 eligible participants; see Fig. 1.

**Fig. 2:**
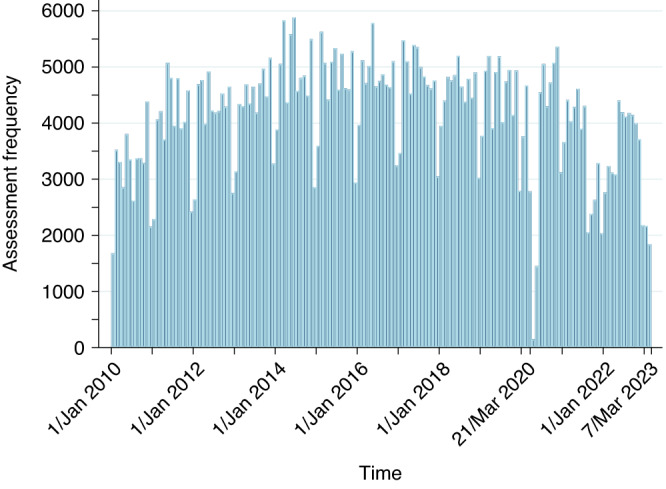
Histogram of monthly child B4SC assessment frequencies over the study period 1 January 2010 and 7 March 2023, inclusive.

Figure 2 presents a histogram of the monthly child B4SC assessment frequencies over the study period. Clear monthly patterns are evident, with December and January having relatively fewer assessments. There was also a dramatic decrease in assessments immediately post-21 March 2020 (when the COVID-19 alert levels were announced), followed by fewer assessments in the post-COVID-19 period.

### Sociodemographic characteristics

The sociodemographic characteristics of children included in the eligible sample is presented in Table 1. This eligible sample had a mean age of 4.4 years (standard deviation: 0.3 years), 320,296 (48.8%) were female, and 156,271 (24.0%) lived in the most deprived quintile. European ethnic identification was most common (*n* = 410,188; 62.7%), followed by Māori (*n* = 152,761; 23.4%), Asian (*n* = 99,573; 15.2%), and Pacific (*n* = 82,044; 12.5%) from the 654,205 participants with valid ethnicity values. Across these groups, 89,038 (13.6%) identified with two, and 10,296 (1.6%) identified with three different ethnicities.

**Table 1:** Sociodemographic characteristics of 656,039 children included in the eligible sample, together with breakdowns by selected periods.

Characteristic	Overall		Ave. Period -3 to-1		Period 1		Period 2		Period 3	
	(*N* = 656,039) *n* (%)		(*N* = 54,327) *n* (%)		(*N* = 44,368) *n* (%)		(*N* = 38,809) *n* (%)		(*N* = 39,681) *n* (%)	
*Age (years): mean (SD)*	4.4	(0.3)	4.3	(0.3)	4.4	(0.3)	4.4	(0.3)	4.5	(0.3)
*Sex*										
Female	320,296	(48.8)	26,479	(48.7)	21,731	(49.0)	18,943	(48.8)	19,499	(49.1)
Male	335,743	(51.2)	27,848	(51.3)	22,637	(51.0)	19,866	(51.2)	20,182	(50.9)
*Ethnicity*^a^										
Māori	152,761	(23.4)	12,995	(24.0)	10,383	(23.5)	10,014	(25.9)	10,027	(25.3)
Pacific	82,044	(12.5)	7333	(13.5)	5535	(12.5)	5096	(13.2)	5691	(14.4)
Asian	99,573	(15.2)	10,175	(18.8)	9224	(20.8)	7494	(19.3)	7888	(19.9)
MELAA^b^	13,242	(2.0)	1302	(2.4)	1212	(2.7)	1063	(2.7)	993	(2.5)
European	410,188	(62.7)	33,240	(61.3)	27,361	(61.8)	25,347	(65.4)	24,788	(62.6)
Other	6027	(0.9)	368	(0.7)	323	(0.7)	217	(0.6)	193	(0.5)
*Level of deprivation*^c^										
1 (least deprived)	127,701	(19.6)	10,649	(19.7)	8897	(20.2)	7927	(20.6)	7624	(19.4)
2	121,430	(18.7)	10,084	(18.6)	8592	(19.5)	7210	(18.7)	7314	(18.6)
3	120,614	(18.5)	10,086	(18.6)	8259	(18.7)	7115	(18.5)	7401	(18.8)
4	124,605	(19.2)	10,197	(18.8)	8452	(19.1)	7316	(19.0)	7689	(19.5)
5 (most deprived)	156,271	(24.0)	13,129	(24.2)	9949	(22.5)	8961	(23.3)	9370	(23.8)
*Region*										
Northern	245,789	(37.5)	21,064	(38.8)	15,923	(35.9)	11,940	(30.8)	13,883	(35.0)
Midlands	138,830	(21.2)	11,512	(21.2)	9425	(21.2)	8483	(21.9)	8366	(21.1)
Central	126,393	(19.3)	10,179	(18.7)	8006	(18.0)	8292	(21.4)	7141	(18.0)
Southern	145,027	(22.1)	11,571	(21.3)	11,014	(24.8)	10,094	(26.0)	10,291	(25.9)

^a^1834 (0.3%) values missing or unknown; ^b^MELAA denotes Middle Eastern/Latin American/African ethnicity; ^c^5418 (0.8%) values missing or unknown; Period –3 to –1 covers 21 March 2017–20 March 2020; Period 1 covers 21 March 2020–20 March 2021; Period 2 covers 21 March 2021–20 March 2022; and Period 3 covers 21 March 2022–7 March 2023.

### Body mass index (BMI)

**Fig. 3:**
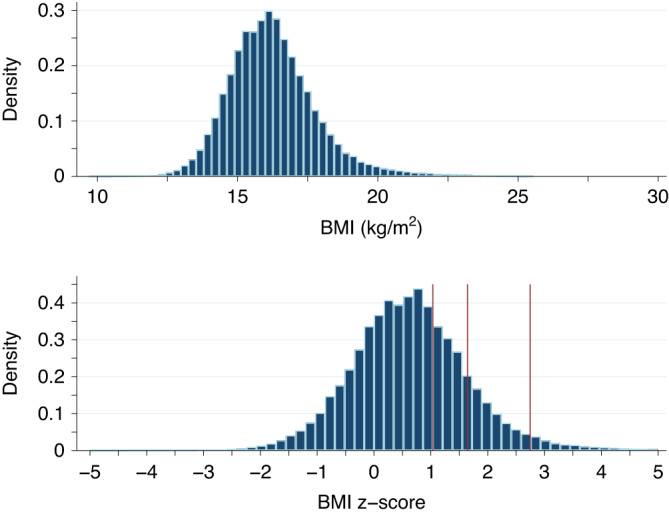
Histograms of BMI (top panel) and BMI z-scores (bottom panel) for 656,039 children with valid biological plausible values. The red vertical lines give the 85^th^ (BMI z-score = 1.036), 95^th^ (BMI z-score = 1645), and 99.7th (BMI z-score = 2.748) percentiles respectively.

Figure 3 presents histograms of both the BMI and BMI z-scores for the eligible participants. When applying BMI z-score classifications, 210,492 (32.1%) participants were defined being overweight, 95,196 (14.5%) as being obese, and 19,926 (3.0%) as being extremely obese.

### Crude analyses

**Fig. 4:**
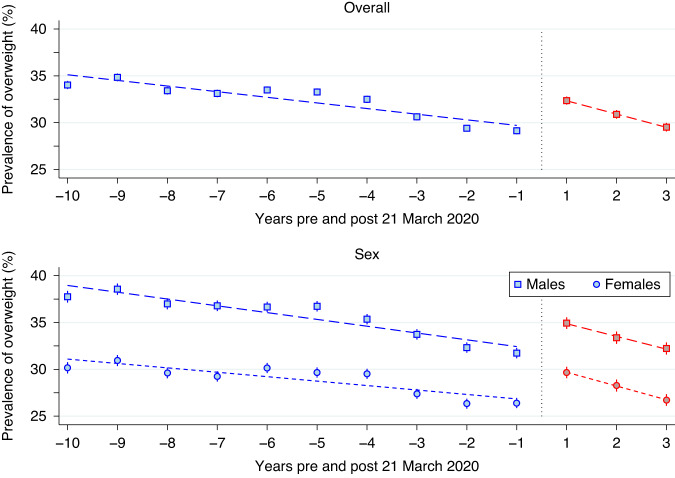
Scatter-plot of overweight prevalence estimates pre (blue) and post (red) 21 March 2020, together with the piece-wise linear regression estimates (dashed line), weighted by participant numbers, for the overall sample (top panel) and stratified by sex (bottom panel). The overall prevalence estimates were denoted by squares, whereas squares and circles were used to denote these estimates for males and females, respectively.

**Fig. 5:**
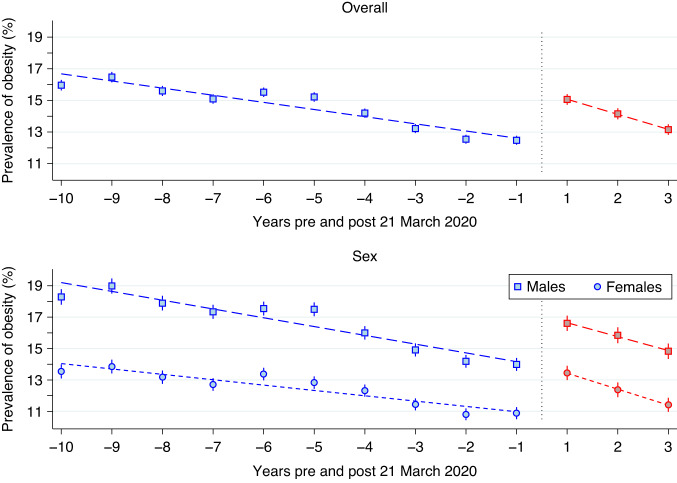
Scatter plot of obesity prevalence. Scatter plot of obesity prevalence pre (blue) and post (red) 21 March 2020, together with the piece-wise linear regression estimates (dashed line), weighted by participant numbers, for the overall sample (top panel) and stratified by sex (bottom panel).

**Fig. 6:**
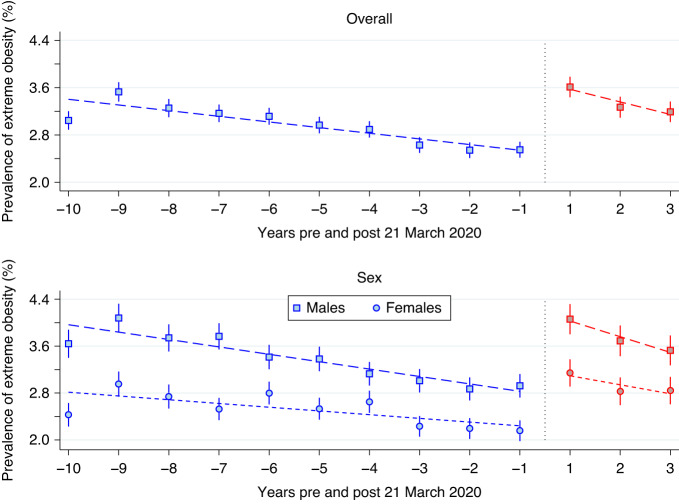
Scatter plot of extreme obesity prevalence. Scatter plot of extreme obesity prevalence pre (blue) and post (red) 21 March 2020, together with the piece-wise linear regression estimates (dashed line), weighted by participant numbers, for the overall sample (top panel) and stratified by sex (bottom panel). The overall prevalence estimates were denoted by squares, whereas squares and circles were used to denote these estimates for males and females, respectively.

Figures 4–6 present scatterplots of yearly overweight, obese, and extreme obese prevalence estimates pre (blue) and post (red) 21 March 2020 for the overall sample and stratified by sex. Super-imposed on these figures are the piece-wise linear regression estimates, weighted by participant assessment numbers, with change point constrained at 21 March 2020. In each of these scatterplots, female prevalence estimates were substantially lower than their male counterparts at all time points. Also, notable significant trends were evident. When considering overweight, in the pre-21 March 2020 period, the overall prevalence decreased by an absolute average of 0.6% (*p* < 0.001) per year to an estimated rate of 29.1% (95% CI: 28.8%, 29.5%) in period –1 (the 21 March 2019–20 March 2020 period). However, in the following period 1 (the 21 March 2020–20 March 2021 period) this estimated prevalence increased to 32.4% (95% CI: 31.9%, 32.8%), a difference that was significant (*p* < 0.001). In the post-21 March 2020 three-year period, the overall prevalence decreased by an average of 1.4% (*p* < 0.001) per year. By period 3 (21 March 2022–7 March 2023) the overall overweight prevalence was estimated at 29.5% (95% CI: 29.1%, 30.0%). The extent of this post-21 March 2020 decrease meant that the overall estimated prevalence in period –1 was no different to period 3 (*p* = 0.21). Similar patterns in the overweight prevalence existed when stratified by sex. The period -1 estimate of overweight was 31.7% (95% CI: 31.2%, 32.3%) for males and 26.4% (95% CI: 25.9%, 26.9%) for females, not significantly different from the period 3 estimates of 32.2% (95% CI: 31.6%, 32.9%; *p* = 0.26) and 26.7% (95% CI: 26.1%, 27.3%; *p* = 0.44) respectively.

For obesity, in the pre-21 March 2020 period, the overall prevalence decreased by an absolute average of 0.5% (*p* < 0.001) per year to an estimated rate of 12.5% (95% CI: 12.2%, 12.8%) in period –1. However, in period 1 this estimated prevalence increased to 15.1% (95% CI: 14.7%, 15.4%), a difference that was significant (*p* < 0.001). In the post-21 March 2020 three-year period, the overall prevalence decreased by an average of 1.0% (*p* < 0.001) per year. By period 3 the overall obesity prevalence was estimated at 13.2% (95% CI: 12.8%, 13.5%). While the post-21 March 2020 decline was relatively rapid, the period 3 prevalence estimates still remained statistically higher than the pre-21 March levels (*p* = 0.003). When stratified by sex, the period –1 estimate of obesity for males was 14.0% (95% CI: 13.6%, 14.4%), significantly lower than the period 3 estimate of 14.8% (95% CI: 14.3%, 15.3%; *p* = 0.01). However, the period -1 estimate of obesity for females was 10.9% (95% CI: 10.5%, 11.3%), not significantly different from the period 3 estimates of 11.4% (95% CI: 11.0%, 11.9%; *p* = 0.08).

When considering extreme obesity, in the pre-21 March 2020 period, the overall prevalence decreased by an absolute average of 0.13% (*p* < 0.001) per year to an estimated rate of 2.5% (95% CI: 2.4%, 2.7%) in period –1. However, in period 1 this estimated prevalence increased to 3.6% (95% CI: 3.4%, 3.8%), a difference that was significant (*p* < 0.001). In the post-21 March 2020 three-year period, the overall prevalence decreased by an average of 0.26% (*p* < 0.001) per year. By period 3 the overall obesity prevalence was estimated at 3.2% (95% CI: 3.0%, 3.4%). While the post-21 March 2020 decline was also relatively rapid, the period 3 prevalence estimates still remained statistically higher than the pre-21 March levels (*p* < 0.001). When stratified by sex, the period -1 estimate of obesity for males was 2.9% (95% CI: 2.7%, 3.1%), significantly lower than the period 3 estimate of 3.5% (95% CI: 3.3%, 3.8%; *p* < 0.001). The period –1 estimate of obesity for females was 2.2% (95% CI: 2.0%, 2.3%), also significantly lower than the period 3 estimates of 2.8% (95% CI: 2.6%, 3.1%; *p* < 0.001).

Figures S1–S3 within the supplementary materials present bubble plots of overweight, obese, and extreme obese prevalence estimates in the year prior to 21 March 2020 (period “–1”) and in the period 21 March 2022–7 March 2023 (period “3”), weighted by participant numbers, over deprivation index and major ethnic groups, stratified by sex. For overweight prevalence, there was no difference between periods in deprivation index or major ethnic groups for males or females (all *p* > 0.05). No difference in obese prevalence estimates was also found between periods in deprivation index (*p* = 0.11) or major ethnic groups (*p* = 0.11) in females. However, obese prevalence estimates remained significantly higher in period “3” compared to period “–1” for deprivation index (*p* = 0.01) and major ethnic groups (*p* = 0.006) for males. Conversely, for extreme obesity, period 3 prevalence estimates were significantly higher than period -1 estimates in deprivation index and among major ethnic groups for both males and females (all *p* < 0.001).

### Changes in assessments and sociodemographic profiles over the COVID-19 period

In the three years prior to 21 March 2020, there was an average of 54,327 eligible assessments per year. However, this reduced to 44,389 (81.7%) assessment in period 1, 38,809 (71.4%) in period 2, and 39,681 (76.0% when accounting for the slightly shorter interval length) in period 3. Table 1 also gives the sociodemographic characteristics of children included in the eligible sample for these periods. In addition to the fewer assessments undertaken in the post-21 March 2020 period, the sociodemographic profiles changed over time. Compared to the 3-year average in the pre-21 March 2020 period, few assessments were undertaken in the Northern region (especially in period 2), the average age at assessment appeared to increase, and those in the most deprived areas appeared less likely to have an assessment. When comparing the average period –3 to –1 profiles to those in period 3, the increase in age was significant (*p* < 0.001), as was the increase in proportion with Māori, Pacific, Asian, and European ethnic identifications (all *p* < 0.001). Conversely, there was a significant decrease in those in the Other ethnic category (*p* < 0.001) and no change among those within the MELAA (*p* = 0.22) group. There also continued to be a significant difference in assessment proportions by region (*p* < 0.001) and level of deprivation (*p* = 0.01) between periods, but not in sex (*p* = 0.15).

### Adjusted analyses

Due to the changing overweight, obesity and extreme obesity prevalence over time, together with the change in sociodemographic profile, the final adjusted analyses compared period –1 to period 3 to establish whether the differences noted the in crude analysis above might be explained by a shift in profiles. Unadjusted and adjusted estimated prevalence ratios (PRs), together with their associated 95% confidence intervals (CIs), from the modified Poisson regression models of overweight, obesity, and extreme obesity between period -1 and period 3, stratified by sex, appears in Table 2. When adjusting for the difference in sociodemographic profiles between periods, the estimated PRs dampened, shrinking towards the null value. The rates in overweight and obesity for both females and males were no different between periods (all *p* > 0.05) in the adjusted analyses. However, PR estimates for extreme obesity remained significantly higher for both females and males between periods (both *p* < 0.05); see Table 2.

**Table 2:** Unadjusted and adjusted estimated prevalence ratios (PRs), together with their associated 95% confidence intervals (CIs), from the modified Poison regression models of overweight, obesity, and extreme obesity between period -1 (assessments undertaken between 21 March 2019–20 March 2020) and period 3 (assessments undertaken between 21 March 2022–7 March 2023), stratified by sex.

	Females				Males			
	Unadjusted		Adjusted*		Unadjusted		Adjusted*	
	PR	(95% CI)	PR	(95% CI)	PR	(95% CI)	PR	(95% CI)
*Overweight*								
Period –1	1	(Reference)	1	(Reference)	1	(Reference)	1	(Reference)
Period 3	1.012	(0.981, 1.044)	1.023	(0.991, 1.057)	1.015	(0.989, 1.042)	1.020	(0.993, 1.049)
*Obesity*								
Period –1	1	(Reference)	1	(Reference)	1	(Reference)	1	(Reference)
Period 3	1.049	(0.995, 1.105)	1.018	(0.964, 1.076)	1.060	(1.014, 1.108)	1.046	(0.999, 1.094)
*Extreme obesity*								
Period –1	1	(Reference)	1	(Reference)	1	(Reference)	1	(Reference)
Period 3	1.317	(1.173, 1.480)	1.204	(1.065, 1.361)	1.207	(1.093, 1.333)	1.126	(1.014, 1.250)

*adjusted for age, ethnicity, level of deprivation, region, and all associated two-factor interactions of these terms.

## Discussion

In ANZ, the patterns of decreasing overweight, obesity, extreme obesity prevalence previously reported over the 2010–2016^11^ and 2017–2019^10^ periods continued until 21 March 2020, when COVID-19 restrictions were implemented.^19^ Similar pre-pandemic patterns have been reported elsewhere, such as in the United States of America (USA)^27^ and the United Kingdom (UK).^28^ However, in the year after COVID-19 restrictions were introduced, significant increases in overweight, obesity, extreme obesity prevalence estimates were observed overall and within sex-specific stratifications, as also reported within Sweden, USA and UK.^28–30^ These elevated post-COVID-19 prevalence estimates then appeared to decrease relatively rapidly over time compared to pre-COVID-19 patterns. So much so, that the prevalence of overweight and obesity in females and overweight in males were no different in period -1 compared to period 3. And when adjusting for changing B4SC assessment sociodemographic profiles, the prevalence of obesity in males was also no different between these periods.

In crude and adjusted analyses, the prevalence of extreme obesity also peaked in period 1 and declined thereafter, similar to the UK.^28^ However, the prevalence estimates in period 3 continued to be significantly higher when compared to period -1, with adjusted PR = 1.204 (95% CI: 1.065, 1.361) for females and 1.126 (95% CI: 1.014, 1.250) for males. Pacific and Māori females and Pacific and Asian males had relatively higher increases in extreme obesity than their counterparts, as did females living within the most deprived areas and males in the highest two levels of deprivation. It is possible that the behavioural, lifestyle and economic changes associated with ANZ’s COVID-19 restrictions disproportionately affected a small subpopulation that have caused an enduring elevation of extreme obesity rates. The delay in lifting restrictions and re-opening services may also have played a contributing role. However, it is interesting to note that overweight and obesity prevalence also increased in 4-year-old Swedish children during the COVID-19 pandemic; a country which never underwent a formal lockdown.^29^

Approximately two-thirds of ANZ’s Pacific population live in Auckland, the nation’s most expensive city for housing and cost of living. Pacific people also carry a disproportionately high economic and health burden compared to other ethnic groups. Auckland experienced the most severe and prolonged lockdown restrictions within the country which likely exacerbated the behavioural, lifestyle and economic factors associated with increased obesity risk. Together with lengthy delays to service provision, these accumulated risks may explain the elevated extreme obesity prevalence estimates among Pacific children. A geospatial study investigating regional effects and differences over time is needed to better understand these mechanisms. It is also possible that the elevated pattern of extreme obesity prevalence will dampen but over a longer timeframe. Alternatively, it is possible that unmeasured confounders or changes in the sociodemographic assessment profiles have biased the estimated prevalence reported here.^31,32^

Along with sex and level of deprivation, ethnicity was associated with higher overweight, obesity, and extreme obesity prevalence rates overall and with higher extreme obesity prevalence estimates post-COVID-19. These pre-pandemic inequities have been described before.^10,11^ We assert that they are predominantly, if not entirely, due to social and commercial determinants of health, together with detrimental and enduring colonisation and immigration histories, rather than any intrinsic cultural or ethnic differences.^33–35^ Largely through economic constraints, Māori and Pacific children are more likely to reside in obesogenic environments targeted by an aggressive and largely unregulated marketing of unhealthy products towards them and their parents.^36,37^ Evidence for successful interventions is scant, and require greater consideration of cultural values and beliefs, community engagement, and exclusive targeting and tailoring to Māori and Pacific children and their families.^38^ It is noteworthy that, although relatively high, the difference in overweight or obesity prevalence for Māori and Pacific 4-year-old children pre and post-COVID-19 did not worsen. Better still would be a reduction in these ethnic inequalities.

This study has a number of strengths, which include a large, near national sample with high population coverage pre-COVID-19, reliable and valid anthropometric measurements,^15^ and an analysis using BMI z-scores derived from WHO and UNICEF defined thresholds.^16^ Important sociodemographic variable were also incorporated, and contemporary methods of analysis and reporting undertaken.^23–25^ However, the study also had salient weaknesses. Despite using a methodology jointly recommended by WHO and UNICEF,^16^ and used widely before, including on 4-year-old children’s B4SC data in ANZ,^11^ we acknowledge that debate remains about what measures and thresholds to best define excess body weight in younger children.^39^ Moreover, based on a study of 1676 females aged 5-16 years, it has been asserted that Māori and Pacific children have different body compositions for a given BMI (such as higher levels of lean body muscle), as compared with other ethnic groups in ANZ.^40^ Universal rather than ethnic-specific thresholds may thus contribute to an overestimation of overweight and obesity in these populations. The cross-sectional design of this study limits any temporal investigations or causal assertions. A study longitudinally tracking body size over time, capturing the pre-and post-COVID-19 period, would provide higher quality evidence.^7^ Also, the study design provides no insight into the outcome for the excess of children with increased overweight, obesity or extreme obesity in the post-COVID-19 period. If this excess was primarily a result of temporary changes to behaviours and lifestyles, then re-established patterns may lead to body size reductions in these children. Another notable feature of these data was that the post-COVID-19 population coverage was considerably smaller with differential uptake by parents and caregivers, introducing potentially increased non-sampling bias.^32^ If non-participation was significantly associated with higher (or lower) obesity prevalence then the resultant presented estimates will systemically underestimate (or overestimate) the underlying rates. Adjusted analyses were undertaken in an effort to mitigate this bias. However, unmeasured confounding variables can also result in substantial bias in the estimated exposure-outcome.^31^ Unmeasured and unadjusted confounding may partially explain the excess levels of extreme obesity observed here. Deprivation was measured in 2006 and 2013 using an area-level combination and weighting of variables.^21^ This geospatial area-based variable may introduce bias through unaccounted clustering and, concordantly, the ecological fallacy. However, the large number of meshblocks, together with their rigorous derivation, likely mitigates the effect of this bias. Finally, the post-COVID-19 period was relatively short – just under 3 years – and future research is needed to understand how this interruption affected the pre-COVID-19 decreasing trends across all excess body size groups, and the inequity between various population subgroups.

## Conclusion

COVID-19 and the associated restrictions appeared to effect overweight, obesity and extreme obesity prevalence in ANZ’s 4-year old children. The pre-COVID-19 year-on-year decline was halted; indeed, there appeared that a substantial and significant short-term increase. However, the most recent data suggest that the prevalence of overweight and obesity have returned to pre-COVID-19 levels. While this represents a hiatus in pre-COVID-19 decreasing trends, future research is needed to determine the medium-term trajectories and whether pre-COVID-19 decreases return.

### Supplementary information

Supplementary materials

## Data Availability

The data that support the findings of this study are available from the Ministry of Health but restrictions apply to the availability of these data, which were used under license for the current study, and so are not publicly available. Data are however available from the authors upon reasonable request and with permission from the Ministry of Health.
