# Supplementary material for: COVID-19 impact on overweight and obesity rates in Aotearoa | New Zealand 4-year-old children
Source: Pediatr Res. 2024 Jan 18;95(6):1649–57. doi: 10.1038/s41390-024-03025-6 (PMC11126386; doi:10.1038/s41390-024-03025-6)

Supplementary materials for:

COVID-19 impact on overweight and obesity rates in Aotearoa | New Zealand 4-year-old children

---

Figure S1. Bubbleplot of overweight prevalence in the year prior to 21 March 2020 (period "-1"; blue) and in the period 21 March 2022 – 7 March 2023 (period "3"; red), weighted by participant numbers, over deprivation index and major ethnic groups, stratified by sex.

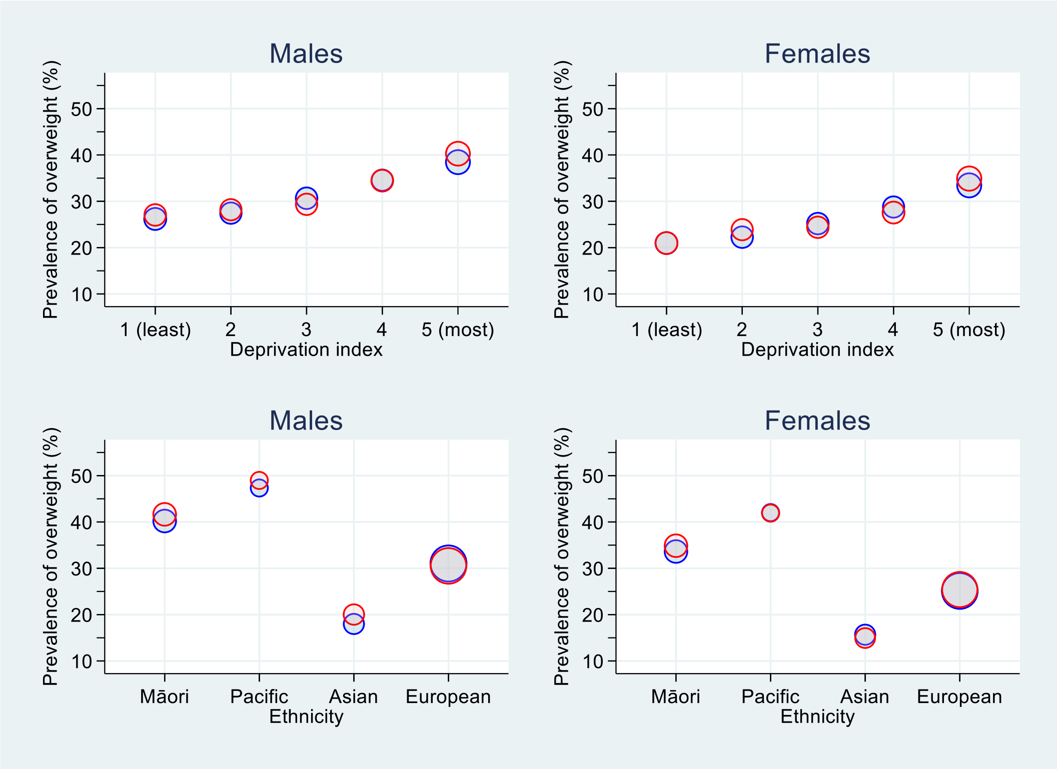

Figure S2. Bubbleplot of obesity prevalence in the year prior to 21 March 2020 (period "-1"; blue) and in the period 21 March 2022 – 7 March 2023 (period "3"; red), weighted by participant numbers, over deprivation index and major ethnic groups, stratified by sex.

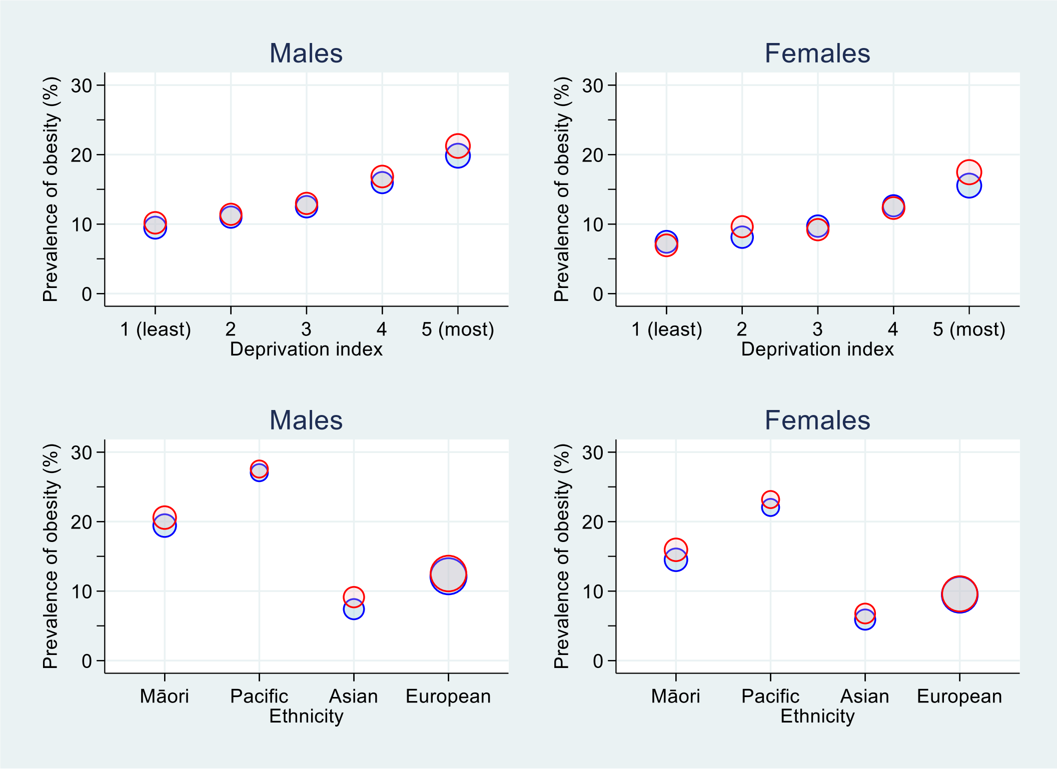

Figure S3. Bubbleplot of extreme obesity prevalence in the year prior to 21 March 2020 (period "-1"; blue) and in the period 21 March 2022 – 7 March 2023 (period "3"; red), weighted by participant numbers, over deprivation index and major ethnic groups, stratified by sex.

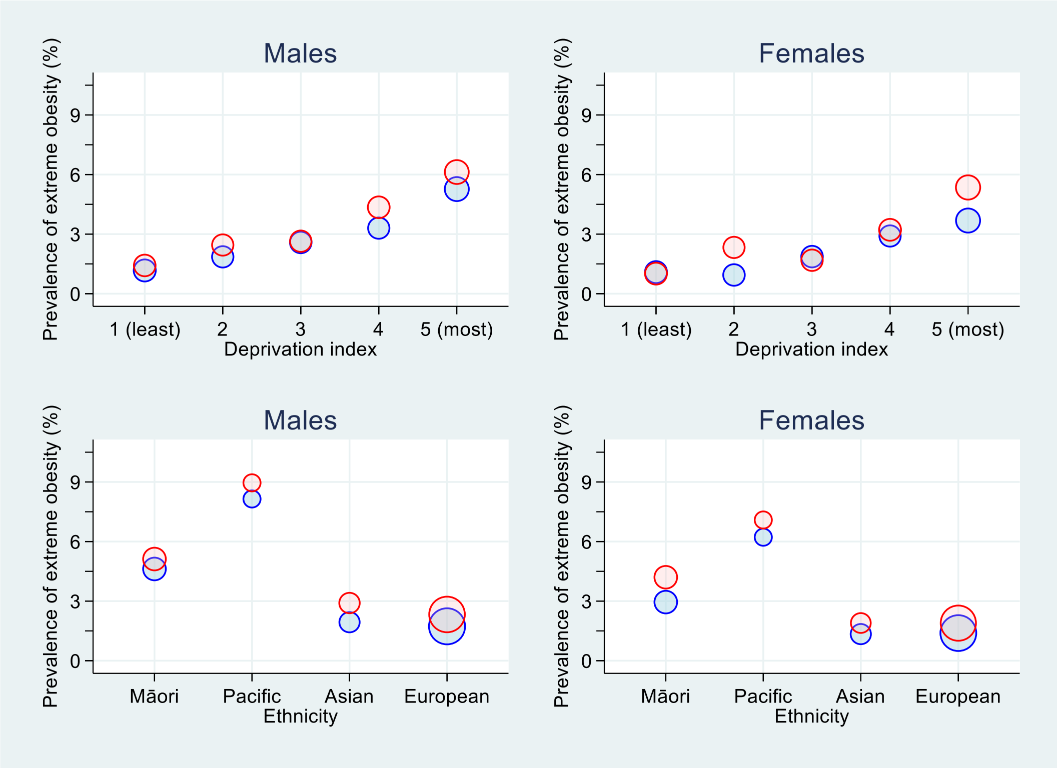

Supplement: Supplementary file 1 — Supplementary materials [file 41390_2024_3025_MOESM1_ESM.pdf]
